# Supplementary material for: A Systematic Review of Genetic Polymorphisms Associated with Binge Eating Disorder
Source: Nutrients. 2021 Mar 5;13(3):848. doi: 10.3390/nu13030848 (PMC7999791; doi:10.3390/nu13030848)
Supplement: Supplementary file 1 [file nutrients-13-00848-s001.pdf]

Table S1. Newcastle-Ottawa quality rating scale (NOS) for non randomised studies included

	Selection				Comparability	Exposure			
**Article**	1	2	3	4	1	1	2	3	**Tot ☆**
Cameron et al (2019) [5]	a ☆	a ☆	a ☆	a ☆	a ☆ (age); b ☆ (BMI)	a ☆	a ☆	a ☆	8
Monteleone et al. (2006) [9]	a ☆	a ☆	c	a ☆	a ☆ (sex)	a ☆	a ☆	b	6
Burnet et al. (1999) [10]	a ☆	a ☆	a ☆	a ☆	a ☆ (age); b ☆ (sex)	a ☆	a ☆	b	8
Ricca et al. (2002) [11]	a ☆	b	c	a ☆	a ☆ (age); b ☆ (BMI)	a ☆	a ☆	b	6
Ceccarini et al. (2020) [12]	a ☆	a ☆	a ☆	a ☆		a ☆	a ☆	b	6
Gonzalez et al. (2019) [15]	a ☆	a ☆	c	b		a ☆	b	b	3
Davis et al. (2007) [16]	a ☆	a ☆	a ☆	a ☆	a ☆ (age)	a ☆	a ☆	b	7
Leehr et al. (2016) [17]	a ☆	b	c	a ☆	a ☆ (age); b ☆ (BMI)	a ☆	a ☆	b	6
Kindler et al. (2011) [18]	a ☆	a ☆	c	a ☆	a ☆ (age); b ☆ (sex)	a ☆	a ☆	b	7
Monteleone et al. (2008) [19]	a ☆	a ☆	a ☆	a ☆	a ☆ (sex)	a ☆	a ☆	b	7
Tortorella et al. (2005) [20]	a ☆	b	c	a ☆	a ☆ (age)	a ☆	a ☆	b	5
Monteleone et al. (2008) [21]	a ☆	a ☆	c	a ☆	a ☆ (sex)	a ☆	a ☆	b	6
Palmeira et al. (2019) [24]	a ☆	a ☆	a ☆	a ☆	a ☆ (sex); b ☆ (age)	a ☆	a ☆	b	8
Davis et al. (2008) [25]	a ☆	a ☆	a ☆	a ☆	a ☆ (age); b ☆ (sex)	a ☆	a ☆	b	8
Davis et al. (2009) [26]	a ☆	a ☆	a ☆	a ☆	a ☆ (age); b ☆ (sex)	a ☆	a ☆	b	8
Davis et al. (2012) [27]	a ☆	a ☆	a ☆	a ☆	a ☆ (age); b ☆ (BMI)	a ☆	a ☆	b	8
Palacios et al. (2018) [28]	a ☆	a ☆	a ☆	a ☆	a ☆ (age); b ☆ (sex)	a ☆	a ☆	b	8
Gervasini et al. (2018) [29]	a ☆	a ☆	c	b		a ☆	b	b	3
Cellini et al. (2010) [30]	a ☆	a ☆	a ☆	a ☆	a ☆ (age)	a ☆	a ☆	b	7
Monteleone et al. (2006) [31]	a ☆	a ☆	c	a ☆	a ☆ (sex)	a ☆	a ☆	b	6
Monteleone et al. (2007) [32]	a ☆	a ☆	a ☆	a ☆	a ☆ (age); a ☆ (sex)	a ☆	a ☆	b	8
Mean					6.62				

BMI: Body Mass Index.
